# Hybrid surgery with PEEK rods for lumbar degenerative diseases: a 2-year follow-up study

**DOI:** 10.1186/s12891-021-04895-1

**Published:** 2022-01-03

**Authors:** Yao Zhao, Beiyu Xu, Longtao Qi, Chunde Li, Lei Yue, Zhengrong Yu, Shijun Wang, Haolin Sun

**Affiliations:** Department of Orthopaedics, Peking University First Hospital, Xicheng District, Beijing, 100034 China

**Keywords:** Lumbar degenerative diseases, PEEK rods, Hybrid surgery, Adjacent segment diseases

## Abstract

**Background:**

Finite element analyses and biomechanical tests have shown that PEEK rods promote fusion and prevent adjacent segment degeneration. The purpose of this study was to evaluate the effects and complications of hybrid surgery with PEEK rods in lumbar degenerative diseases.

**Methods:**

From January 2015-December 2017, 28 patients who underwent lumbar posterior hybrid surgery with PEEK rods were included in the study. The patients were diagnosed with lumbar disc herniation, lumbar spinal stenosis, or degenerative grade I spondylolisthesis. Before the operation and at the last follow-up, the patients completed lumbar anteroposterior and lateral X-ray, dynamic X-ray, MRI examinations. In addition, at the last follow-up the patients also completed lumbar CT examinations. The radiographic parameters, clinical visual analog scale (VAS) score and Oswestry disability index (ODI) score were compared.

**Results:**

The average age of the patients was 44.8 ± 12.6 years, and the average follow-up duration was 26.4 ± 3.6 months. The VAS score improved from 6.3 ± 1.6 to 1.0 ± 0.9, and the ODI score decreased from 38.4 ± 10.8 to 6.8 ± 4.6. The fusion rate of the fused segment was 100%. There were no significant changes in the modified Pfirrmann classifications or disc height index for the nonfused segments and the upper adjacent segments from pre- to postoperatively. No cases of screw loosening, broken screws, broken rods or other mechanical complications were found.

**Conclusion:**

Hybrid surgery with PEEK rods for lumbar degenerative diseases can yield good clinical results and effectively reduce the incidence of complications such as adjacent segment diseases.

**Supplementary Information:**

The online version contains supplementary material available at 10.1186/s12891-021-04895-1.

## Background

Lumbar decompression with fusion surgery is currently a common treatment for lumbar degenerative diseases. Titanium rods are widely used and can yield sufficient stability and a high fusion rate. However, due to the large elastic modulus of titanium rods, shortcomings, such as stress shielding in the intervertebral bone graft area and increased stress on adjacent segmental discs and facet joints, can occur, thereby increasing the risk of adjacent segment disease (ASD). The annual incidence rate of ASD has been reported to be 2-3% [1]. A 10-year follow-up study of patients with single-segment lumbar fusion by titanium rods showed that the incidence of radiological ASD was 75%, the incidence of symptomatic ASD was 31%, and the rate of revision surgery due to ASD was 15%, where 77% of revisions were due to cranial degeneration [2]. ASD can cause back pain, radiating pain and numbness in the lower limbs, restrict the patient’s ability to perform activities and reduce their quality of life.

With the development of suitable biomechanical materials, elastic fixation and semirigid fixation have emerged. Elastic fixation, such as the Dynesys system, retains the mobility of the corresponding segment but increases the stress at the screw-bone interface, which increases the risk of screw loosening. Payer et al. [3] used the Dynesys system to treat patients with single-segment degenerative spondylolisthesis and spinal stenosis, and the results showed that it could not effectively prevent the occurrence of ASD. A 5-year follow-up study showed that the incidence of screw loosening was as high as 20% [4].

Semirigid fixation, with systems such as the polyetheretherketone (PEEK) rod system, has been used in lumbar spine surgery since 2007 [5]. The PEEK material has good levels of biocompatibility, nontoxicity, and corrosion resistance. Its elastic modulus is approximately 3.2 GPa, which is between those of cancellous bone and cortical bone and is significantly lower than the that of titanium rods, which is 114 GPa [6]. Compared with titanium rods, PEEK rods increase the load on the anterior column, allow the fixed segment to move slightly, reduce the stress shielding effect, and reduce the stress on the interface between the bone and screw [7–9]. In addition, PEEK rods are transparent on X-ray fluoroscopy; therefore, the range of artifacts is small during CT and MRI examinations.

Previous clinical studies have shown that PEEK rods can achieve satisfactory results in lumbar short-segment fusion and are not inferior to the current “gold standard” titanium rods [10]. ASD is a common complication of lumbar fusion surgery. The risk factors include an age over 60 years, obesity, preoperative disc and facet joint degeneration, long-segment fusion, and insufficient lumbar lordosis (LL) recovery [11]. In some special cases, such as in patients with significant degeneration of adjacent segments, PEEK rods are more advantageous in theory. Topping-off surgery refers to the use of a stabilizing device above the lumbar fusion segment, which also has a potential preventive effect on proximal ASD [12]. At present, the clinical research on PEEK rods is relatively scarce. This study aimed to determine the clinical efficacy of topping-off hybrid surgery with PEEK rods in treating lumbar degenerative diseases and its impact on ASD.

## Methods

Patients who underwent two-segment hybrid surgery with PEEK rods in our hospital due to lumbar disc herniation between January 2015 and December 2017 were recruited. The research was approved by the Ethics Committee of Peking University First Hospital (No. 2015 [953]). Before surgery, the patients completed lumbar X-ray, dynamic X-ray and MRI examinations. The affected segments (fusion segment) were located at L5/S1 or L4/5 and were treated with decompression and intervertebral fusion. Indications for fusion included: (1) huge disc herniation with prolapse of the annulus fibrosus and cartilage endplate, and serious destruction of intervertebral disc structure; (2) dynamic radiograph showed lumbar instability (slippage≧3 mm); (3) the decompression range exceeded 1 / 3 of the facet joint, or it affected the stability of the lumbar spine; (4) extreme lateral intervertebral disc herniation requiring resection of facet joint. There was significant degeneration in the upper adjacent segments (nonfusion segment), according to findings such as a low signal intensity of the disc on T2 phase images, a high-intensity zone (HIZ) behind the disc, a reduced intervertebral height, lumbar disc herniation without nerve compression, combined with grade 1 stenosis [13] or degenerative spondylolisthesis (Meyerding I), and the segments were fixed without fusion. The exclusion criteria included lumbar spondylolysis, lumbar tumors, spinal deformities, a history of lumbar surgery, and related conditions. At the 2-year follow-up, lumbar X-ray, dynamic X-ray, CT and MRI scans were performed. The VAS scores and ODI scores were recorded pre- and postoperatively.

**Fig. 1:**
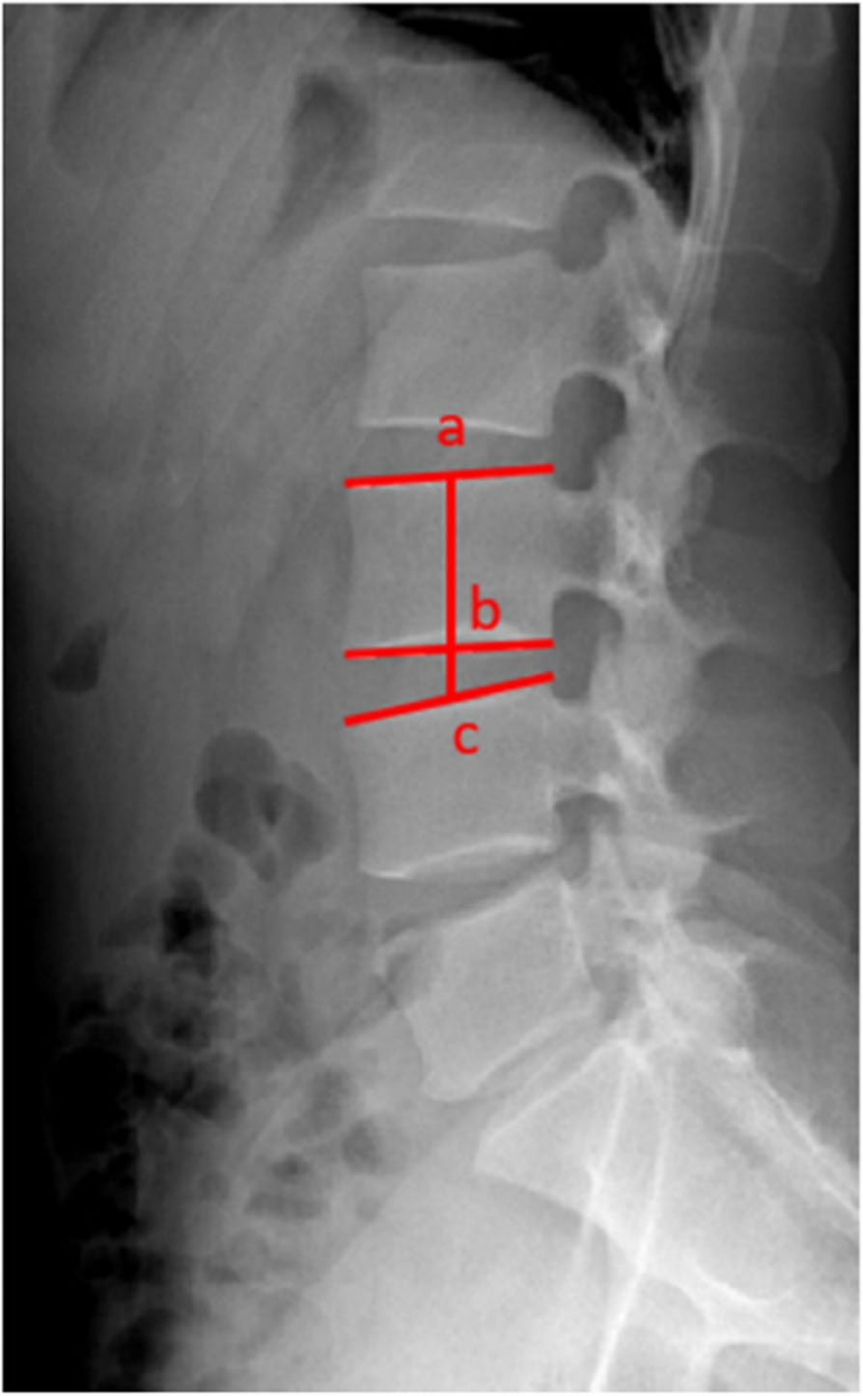
Schematic diagram of the disc height index (DHI). The midpoints of the upper and lower endplates of the upper vertebral body are marked as a and b, respectively, and the midpoint of the upper endplate of the lower vertebral body is marked as c, DHI = bc/ab

The radiological parameters included the preoperative and postoperative values of LL, pelvic incidence (PI), fixed-segment lordosis and range of motion (ROM), nonfusion segment and upper adjacent segment lordosis, disc height index (DHI) (Fig. 1), and modified Pfirrmann classification [14] of the discs.

SPSS version 22.0 (IBM, Armonk, NY, USA) was used for statistical analysis, and the average and standard deviation of each parameter were calculated. The preoperative and postoperative modified Pfirrmann classification were determined using Wilcoxon signed ranks test, and the other parameters were determined using paired-sample t tests, and *p* values < 0.05 were considered to indicate significant differences.

## Results

There were 39 patients who underwent hybrid surgery, 28 of whom completed the 2-year follow-up and were included in this study. The general information of the patients is shown in Table 1. One patient had cerebrospinal fluid leakage due to the adhesion of the intervertebral disc and dural sac during the operation, which was repaired by sutures. Fat liquefaction occurred in 1 patient, and delayed healing occurred in 1 patient due to a superficial wound infection. At the two-year follow-up, the fusion rate was 100%, as evidenced by CT. There were no mechanical complications, such as screw loosening or screw and rod breakage, and no patients required revision surgery due to complications.

**Table 1:** General information of the patients

Age (years)	44.8 ± 12.6 (28-71)
Sex (male/female)	18/10
BMI (kg/m^2^)	25.5 ± 3.5 (20.2-34.3)
Follow-up duration (months)	26.4 ± 3.6 (21-36)
Surgical segments (L3-5/L4-S1)	9/19
Operation time (minute)	155.4 ± 23.0 (110-210)
Intraoperative bleeding (ml)	162.5 ± 64.7 (50-300)

The VAS score improved from 6.3 ± 1.6 to 1.0 ± 0.9 (*p* < 0.05), and the ODI score decreased from 38.4 ± 10.8 to 6.8 ± 4.6 (*p* < 0.05). The magnitudes of improvement in pain and function were significant.

**Fig. 2:**
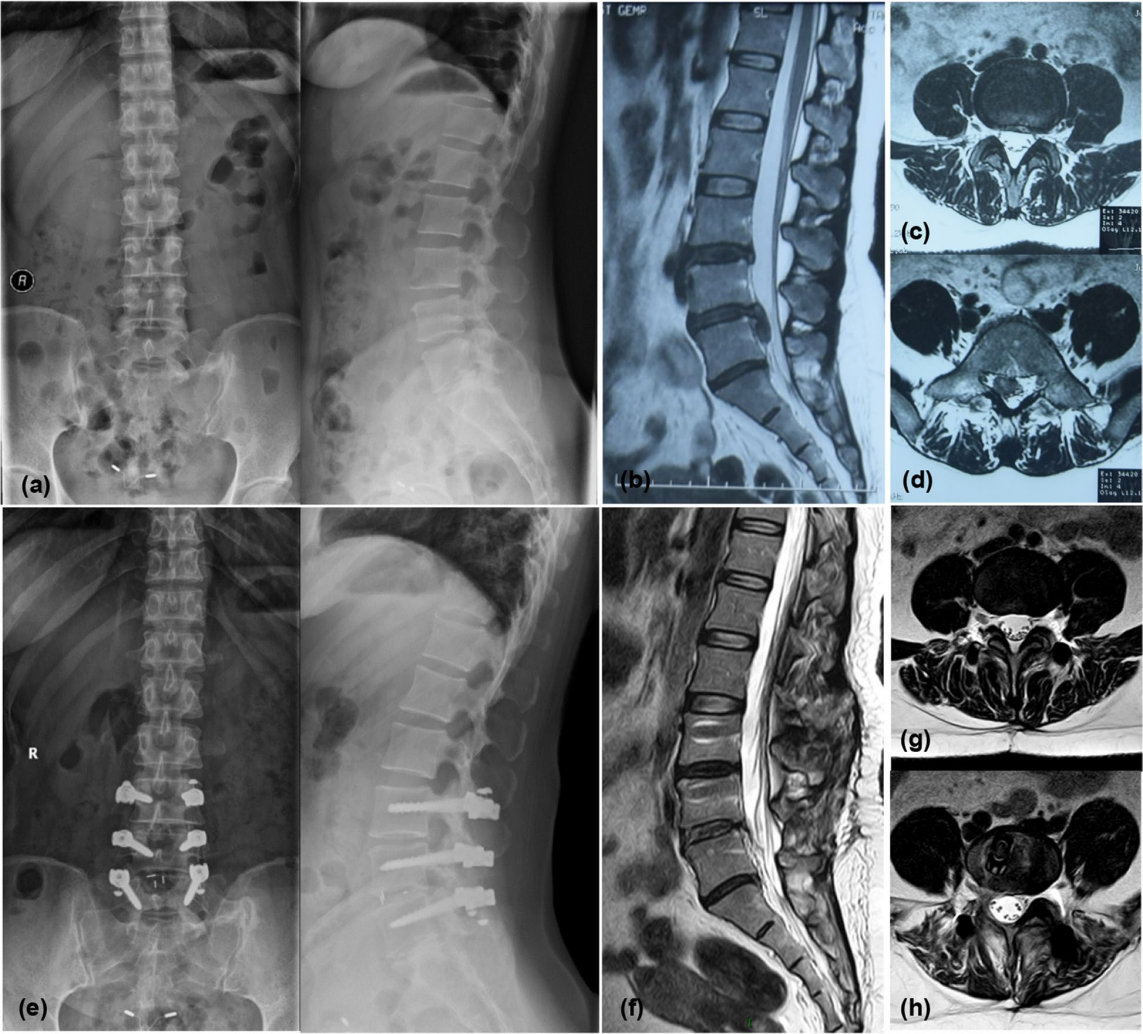
Typical case. The patient was a 40-year-old woman who was diagnosed with lumbar disc herniation (L4/5), cauda equina syndrome, and L3/4 disc degeneration and underwent L3-5 hybrid surgery with PEEK rods. **a** shows the lumbar X-ray before the operation. **b** shows the sagittal-plane MRI image of the lumbar region before the operation.**c**, **d** show the MRI images of the L3/4 and L4/5 sections of the lumbar region before the operation, respectively. **e** shows the lumbar X-ray at the 2-year follow-up. **f** shows the sagittal MRI image of the lumbar region at the 2-year follow-up. **g**, **h** show the MRI images of the L3/4 and L4/5 sections of the lumbar region at the 2-year follow-up, respectively. L3-5 were fixed segments, L3/4 was nonfused segments, the modified Pfirrmann classification of L3/4 was 6 both pre- and postoperatively, and the DHI was 0.28 preoperatively and 0.26 postoperatively. The modified Pfirrmann classification of the upper adjacent segment L2/3 was 2 both pre- and postoperatively, and the DHI was 0.33 preoperatively and 0.35 postoperatively. No significant degeneration was seen

**Fig. 3:**
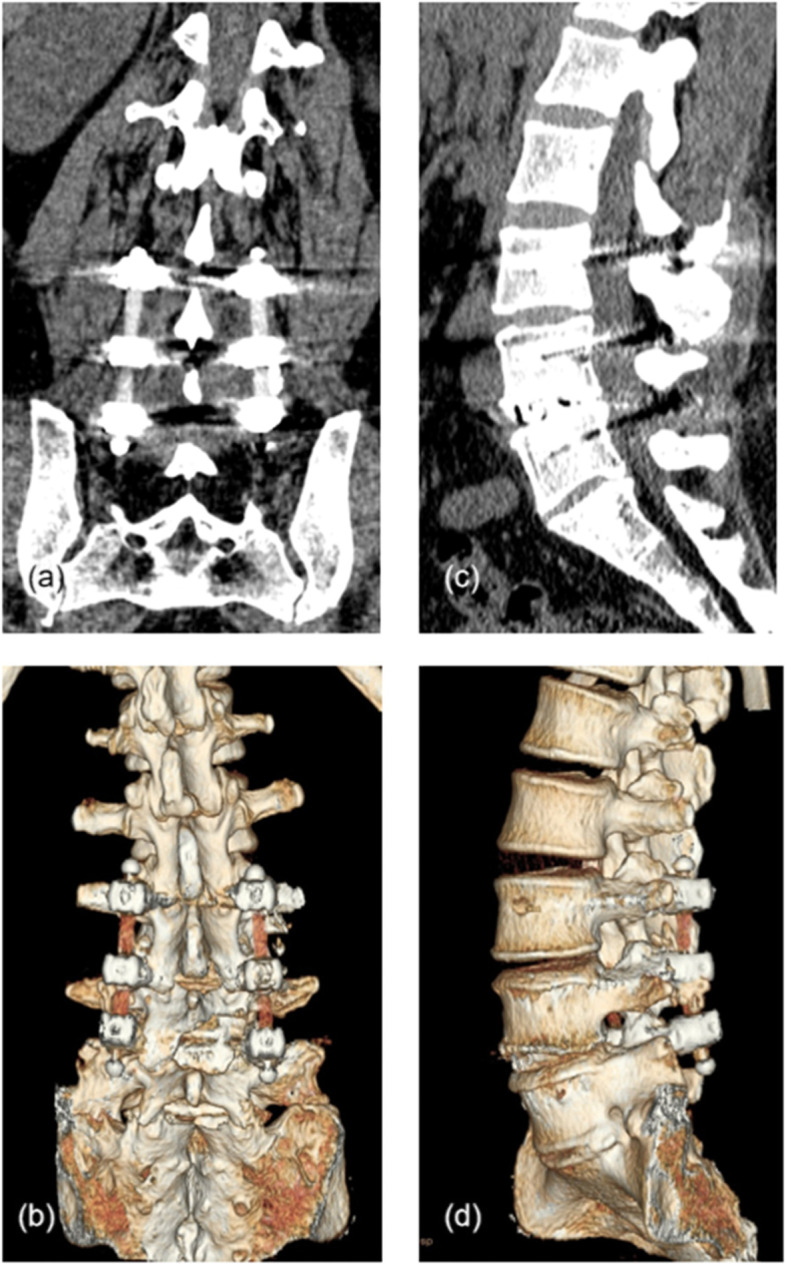
Typical case. **a**, **b** Coronal lumbar CT scan and 3D reconstruction taken at the 2-year follow-up, showing the contours of the bilateral PEEK rods. **c**, **d** Sagittal plane image and 3D reconstruction showing intervertebral fusion was achieved at the L4/5 segment

The comparison of radiological parameters from pre- to postoperatively is shown in Table 2. There were no significant changes in the modified Pfirrmann classifications or DHI of the intervertebral disc at the nonfusion segment and its adjacent segment from preoperatively to the two-year follow-up. The lordosis of the nonfusion segment decreased from an average of 10.5° to 8.3°, and the lordosis of the upper adjacent segment increased from an average of 9.3° to 10.7°. The changes were significant. The fixed-segment ROM significantly decreased from an average of 10.0° to 2.6°. There were no significant changes in fixed-segment lordosis, LL or PI from before to after the operation. The typical case is shown in Figs. 2 and 3.

**Table 2:** Comparison of radiological parameters from pre- to postoperatively

Radiological parameters	Preoperation	2-year follow-up	*P* value
Modified Pfirrmann classification of nonfusion segment	5.0 ± 1.0	4.9 ± 1.1	0.102
DHI of nonfusion segment	0.34 ± 0.07	0.33 ± 0.05	0.233
Lordosis of nonfusion segment (°)	10.5 ± 4.3	8.3 ± 3.0	0.005
Modified Pfirrmann classification of upper adjacent segment	3.3 ± 1.2	3.4 ± 1.2	0.655
DHI of upper adjacent segment	0.34 ± 0.04	0.33 ± 0.04	0.450
Lordosis of upper adjacent segment(°)	9.3 ± 3.6	10.7 ± 3.7	0.010
Lordosis of fixed-segment (°)	27.5 ± 9.3	26.8 ± 6.4	0.612
Lordosis of fixed-segment at flexion position (°)	21.2 ± 9.2	25.3 ± 6.9	0.004
Lordosis of fixed-segment at extension position (°)	31.2 ± 9.4	27.8 ± 7.0	0.015
ROM of fixed-segment (°)	10.0 ± 3.9	2.6 ± 1.2	0.000
LL (°)	43.0 ± 14.6	44.4 ± 11.0	0.411
PI (°)	45.1 ± 9.2	45.8 ± 8.8	0.225

Pearson correlation analysis showed that there was a significant positive correlation between postoperative fixed-segment lordosis and preoperative fixed-segment lordosis and LL (*p* < 0.05), with *r* = 0.695 and 0.558, respectively. There were no significant correlations between fixed-segment mobility and other factors.

## Discussion

Lumbar degenerative disease begins with degeneration of a lumbar intervertebral disc, most commonly at the L4/5 and L5/S1 segments. In clinical practice, multiple disc degeneration is often seen. Due to a decrease in water content and the elasticity of the disc, the stress on the facet joint becomes abnormal, and the stability of the lumbar spine decreases. It is still controversial whether to perform fusion surgery for lumbar degenerative spondylolisthesis. Recently, the NORDSTEN-DS trial showed that decompression alone was noninferior to decompression with instrumented fusion over a period of 2 years. Reoperation occurred somewhat more often in the decompression-alone group than in the fusion group [15]. However, the American SLIP trial showed significantly better results for fusion. The patients in this study had huge disc herniation and lumbar instability at affected segment, we preferred fusion surgery [16]. Titanium rods are the most widely used rods for fusion. However, long-term follow-up results have shown that the risk of adjacent segment degeneration is as high as 75% [2]. The patients had multisegment degeneration, and the average body mass index (BMI) was 25.5 kg/m^2^. Because obesity is also a risk factor for ASD [11], for these patients, we tended to choose hybrid surgery with PEEK rods to reduce the risk of ASD. Since 18 patients in this study underwent L4-S1 fixation and ASD mostly occurred in the upper adjacent segment [2], this article mainly evaluated cases in the upper adjacent segment.

The modified Pfirrmann classification of the nonfusion segment was 5.0 preoperatively and 4.9 at the 2-year follow-up, and the DHI was 0.34 preoperatively and 0.33 at the 2-year follow-up, showing these parameters hardly changed. Mesbah et al. [17] simulated L4/5 fusion with titanium rods and L3-5 hybridization with PEEK rods with finite element analysis, and the results showed that PEEK rods can significantly reduce the pressure of the L3/4 disc during flexion, extension, and lateral flexion. The titanium rods increased the stress on the L3/4 facet joints by 152%, while the PEEK rods reduced the stress on the L3/4 facet joints by 25%. Because the PEEK rods provided support to the nonfusion segment, they reduced the stress on the disc and the facet joint, so these rods might protect the degenerated segment.

Preoperatively, the modified Pfirrmann classification of the upper adjacent segment was 3.3, and the DHI was 0.34. At the 2-year follow-up, the modified Pfirrmann classification was 3.4, and the DHI was 0.33, showing these parameters hardly changed. Obvious adjacent segment degeneration was not seen. Hsieh et al. [18] established a finite element model of L4/5 fusion with connecting rods of different materials. The results showed that compared with titanium rods, PEEK rods could reduce the stress on the facet joints and disc of the upper adjacent segment and increase the stress on the intervertebral cage and bone graft. De Lure et al. [19] reviewed the data of 30 patients who underwent fusion surgery with PEEK rods, and ASD was not found at the follow-up performed at an average of 18 months postoperatively. In this study, hybrid surgery was performed to form a buffer between the fixed vertebrae and the normal vertebrae and reduce the stress. We believe that it had a helped prevent ASD, and the short-term follow-up results were good.

The biomechanical tests showed that titanium rods and PEEK rods can provide similar levels of stability. Although both types of rods can significantly reduce the mobility of the fixed segment (> 70%), PEEK rods can provide greater all-directional mobility than can titanium rods [20–22]. The finite element analysis showed that compared with titanium rods, PEEK rods can increase the range of flexion and extension, lateral flexion and axial rotation by 1.8-2.1, 7-7.2 and 3.7-3.8 times, respectively [23, 24]. Biswas et al. [24] performed finite element analysis and reported that the mobility of the fixed segment of PEEK rods was 4.2°-6.2° in flexion and 4.2°-1.1° in extension. Huang et al. [25] used the nonfusion technique with PEEK rods to treat lumbar degenerative diseases. There were no significant changes in the height of the disc at the treated segment during the 2-year follow-up period, and the ROM was 1.8°. In this study, the motion of the fixed segment was 2.6°, which confirmed that PEEK rods do allow the fixed segment to move slightly, consistent with the results of finite element analysis.

The restoration of a normal LL curve is very important. Ideally, LL = PI ±9° [26]. In this study, the postoperative LL was 44.4°, which was similar to the postoperative PI value of 45.8°, and the difference was not significant. The lordosis of the fixed segment was 26.8°, which was not significantly different from the preoperative lordosis, but the postoperative lordosis of the nonfused segment was 8.3°, which was 2.2° less than that before the operation, and the difference was significant. Because PEEK rods cannot be bent during the operation, it suggested that PEEK rods are still slightly insufficient in restoring lordosis. The postoperative lordosis of the upper adjacent segment was 10.7°, which was increased by 1.4° to retain the LL. Ogrenci et al. [27] reported the mid-term follow-up results of 172 patients treated with PEEK rods. The average LL was 42.5° preoperatively and 44.0° postoperatively. The authors also believed that PEEK rods are less effective in restoring LL and reconstructing sagittal balance. Therefore, for patients with high PI (> 55°), PEEK rods might have adverse effects on sagittal balance. In the future, PEEK rods should be designed with different radians according to the PI value of the patients to solve this problem.

Under normal physiological conditions, the lumbar disc bore approximately 80% of the stress, and the posterior facet bore 20% of the stress [28]. When the PEEK rods were used, the pressure on the front column was 59%, which was higher than that when the titanium rods were used, which was 55% [29]. Increasing the load on the anterior column can reduce the stress shielding effect and promote bone graft fusion. In this study, the fusion rate of the fused segment was 100%, which is consistent with the findings of Qi et al [30].

No mechanical complications, such as screw loosening or screw and rod breakage, were found in this study. Meta-analysis showed that the fusion rate of PEEK rods was as high as 95.6%, and the incidence rates of screw breakage and screw loosening were 2.6 and 2.0%, respectively [10]. Huang et al. [25] found that in 31 patients, only one patient had a halo zone around the screw on radiography, and the patient did not have clinical symptoms. Lure et al. [19] reported that 1 of 30 patients underwent revision surgery due to screw loosening. In this case, the patient had a flat back deformity, and the fixation length was 4 segments. Ormond et al. [31] reported that the fusion rate of PEEK rods in the treatment of lumbar degenerative diseases was 89.3%, and the revision rate was 19.1%, where 62.5% of the revisions were due to ASD. A possible explanation for this finding is that there were many smokers among the enrolled patients, and the article did not report whether there was degeneration in the adjacent segments before surgery. Krieg et al. [32] reported 322 patients with an average age of 69.1 years who received PEEK rods during topping-off hybrid surgery. Among these patients, 18% developed ASD, and the average time of appearance was 26.5 months after surgery. A total of 21.1% of patients had a halo zone around the screws, and 16.4% of patients required revision surgery. These findings were quite different from ours. The possible reasons are as follows: the average age of the patients in this study was 44.8 years old, and the incidence of ASD is higher in patients over 50 years old [33]. Because the patients were relatively young, the bone condition was better, and the intervertebral fusion rate reached 100%, reducing the risk of screw loosening. In addition, the average number of fixed segments was 3.2in the study by Krieg et al. [19] and 2 in our study. Long segment fixation might also affect ASD [11].

PEEK rods have some elasticity, so the risk of rod breakage was low. Cases of rod breakage have rarely been reported in the literature. Kurtz et al. [34] reported 12 cases of revision surgery, and PEEK rods were retrieved, but none of them were broken. In a mid-term follow-up study reported by Ogrenci et al. [27], rod breakage occurred in 1 of 172 patients. Although PEEK rods are invisible on X-ray images, the contours of the rods are visible on CT images. No cases of broken rods were found in our study.

The VAS score and ODI score significantly improved, which is consistent with the results of previous studies [19, 25, 27]. Sarbello et al. [35] considered that the radiolucency of the PEEK rods might affect the perceptions of the patients. The patients who received PEEK rods tended to have better outcomes. Meta-analysis showed that the postoperative improvement rate of clinical function was 67.4%, which was similar to that associated with titanium rods [10].

This study has the following shortcomings. First, it was a retrospective study and lacked a randomized control group. Second, the sample size was small, and there might be selection bias. In addition, the follow-up period was short. At present, the number of clinical trials on hybrid surgery with PEEK rods is very small, and there is a lack of long-term follow-up results. Additional research is still needed to confirm the clinical efficacy of hybrid surgery with PEEK rods.

## Conclusion

In patients with the appropriate indications, hybrid surgery with PEEK rods could be performed to treat two-segment lumbar degenerative diseases. Compared with titanium rods, PEEK rods can theoretically increase the load on the anterior column of the vertebral body to promote intervertebral fusion and simultaneously reduce the pressure on the discs and articular processes of the upper adjacent segments to reduce the risk of ASD. In this study, there were no cases of ASD, screw loosening, breakage or other mechanical complications at the 2-year follow-up. The clinical symptoms improved satisfactorily. Additional long-term and large-scale clinical studies are needed to confirm the clinical efficacy of PEEK rod hybrid surgery.

## Supplementary Information

**Additional file 1.**

## Data Availability

The datasets analyzed during the current study are available from the corresponding author on reasonable request.

## Abbreviations

PEEK: Polyetheretherketone
MRI: Magnetic Resonance Imaging
CT: Computed Tomography
VAS: Visual Analog Scale
ODI: Oswestry Disability Index
ASD: Adjacent Segment Disease
LL: Lumbar Lordosis
PI: Pelvic Incidence
ROM: Range of Motion
DHI: Disc Height Index
NORDSTEN-DS trial: The Norwegian Degenerative Spondylolisthesis and Spinal Stenosis
SLIP Trial: Spinal Laminectomy versus Instrumented Pedicle Screw Trial
